# Prevalence and determinants of anaemia among pregnant women using biomass fuel in rural Tamil Nadu: a cross-sectional study

**DOI:** 10.1186/s12884-026-08999-1

**Published:** 2026-03-27

**Authors:** Vigneswari Aravindalochanan, Sheela Sinharoy, Gurusamy Thangavel, Naveen Puttaswamy, Sarada Satyamoorthy Garg, Sankar Sambandam, Krishnendu Mukhopadhyay, Durairaj Natesan, Rengaraj Ramasamy, Karthikeyan Rajamani, Priyakumar Natarajan, Meenakshi Sundarem Gopalkrishna, Lindsay M. Jaacks, Kalpana Balakrishnan, Usha Ramakrishnan, Shirin Jabbarzadeh, Barr DB, Thomas Clasen, William Checkley, Jennifer L. Peel, HAPIN Investigators

**Affiliations:** 1 Department of Environmental Health and Engineering, Faculty of Public Health, ICMR Center for Advanced Research on Air Quality, Sri Ramachandra Institute for Higher Education and Research (Deemed University), Chennai, Tamil Nadu India; 2 Department of Environmental Health, Rollins School of Public Health, Emory University, Atlanta, GA USA; 3 Global Academy of Agriculture and Food Systems, The University of Edinburgh, Midlothian, UK; 4 Division of Pulmonary and Critical Care, School of Medicine, Johns Hopkins University, Baltimore, MD USA; 5 Department of Environmental and Radiological Health Sciences, Colorado State University, Fort Collins, CO USA; 6 Department of General Medicine, Saveetha Institute of Medical and Technical Sciences, Saveetha University, Chennai, Tamil Nadu India; 7 Department of Environmental Health Engineering &Faculty of Public Health WHO Collaborating Center for Training Occupational and Environmental Health, Climate and Health, SRU - ICMR Center for Air Quality, Sri Ramachandra Institute of Higher Education & Research (Deemed to be University, Porur, Chennai, 600116 Tamil Nadu India

**Keywords:** Anaemia, Pregnant women, South India, Rural, Household air pollution

## Abstract

**Background:**

Anaemia during pregnancy is a major public health concern in India. Biomass fuel use is common in a few rural areas of Tamil Nadu state and there is growing evidence that its use is associated with anaemia. The aim of this study was to estimate the prevalence of anaemia and to identify the coexisting determinants of anaemia among exclusive biomass-using rural pregnant women.

**Methods:**

We conducted a cross-sectional analysis of baseline data collected from 799 rural pregnant women (gestational age between 9 and 20 weeks) from two districts of Tamil Nadu, India. All study participants used biomass for cooking and were enrolled in a multi-country randomised controlled trial of a liquefied petroleum gas cookstove intervention. Haemoglobin (Hb) was measured in capillary blood using the HemoCue^®^201 point of care device. Hb was categorized as normal (Hb ≥ 11 g/dL), mild anaemia (Hb 10.0–10.9 g/dL), moderate anaemia (Hb 7.0–9.9 g/dL), or severe anaemia (Hb < 7.0 g/dL). Multinomial and binary logistic regressions were used to identify factors associated with anaemia.

**Results:**

Overall, prevalence of any anaemia was 66.7% (95% confidence interval: 63.4–70%) and that of mild anaemia was 31.9% (28.7–35.1%), moderate anaemia 33.7% (30.4–36.9%), and severe anaemia was 1.1% (0.6–2.2%). Body mass index classified as underweight (< 18.5 kg/m^2^), being a multigravida, and no observed hand washing facility in the household were associated with a significantly higher odds of anaemia.

**Conclusion:**

This study population of exclusive biomass using pregnant women had higher rates of anaemia and underweight than that of rural estimates from recent NFHS reports for Tamil Nadu state, indicating the need to improve their overall anthropometric status and anaemia together, emphasizing the need for a transition to cleaner fuel.

**Supplementary Information:**

The online version contains supplementary material available at 10.1186/s12884-026-08999-1.

## Introduction

Maternal and child malnutrition is a leading risk factor for disease, contributing to 11.6% of all annual global disability-adjusted life years [1]. Anaemia during pregnancy is a major public health concern due to its impact on maternal and child health such as increased risk of infection, preterm delivery, low birth weight, infant mortality, and poor growth and development of children [2–4]. Globally, 38% of pregnant women are anaemic [5], with South Asian countries carrying the largest burden.

In India, home to one-fifth of the world’s population, the most recent National Family Health Survey (NFHS-5) conducted in 2019–2021 indicated that 52.2% of pregnant women were anaemic [6]. Notably, this prevalence suggested an increase in anaemia among pregnant women, compared with a prevalence of 50.4% in 2015–2016 (NFHS-4) [7]. Despite widespread iron supplementation programs, the burden of anaemia among pregnant women in India continues to rise. Similar to national estimates, the NFHS-4 to NFHS-5 reports show an increase in anaemia prevalence for pregnant women in Tamil Nadu state as well [6, 7]. Overall, several states in India are not on-track to meet National Nutrition Program (NNM) targets by 2022 or the UN Sustainable Development Goal target for anaemia by 2030 [8].

These findings support the complex aetiology of maternal anaemia, with factors other than micronutrient intake playing a role. A main driver of anaemia among pregnant women is an increased physiological iron requirement during pregnancy, combined with inadequate iron intake and absorption, especially among those who consume a vegetarian diet. Social drivers of anaemia in rural pregnant women include socio-economic conditions, maternal education, age at pregnancy. Environmental drivers of anaemia include poor sanitation conditions, including the practice of open defaecation [9], inadequate access to safe water [10] and use of biomass fuels for cooking [11]. These environmental conditions can lead to chronic systemic inflammation developed by long-term exposure to pathogens and pollutants [12]. Research indicates that particulate matter from biomass combustion causes direct red blood cell damage through oxidative stress and inflammation. Additionally, cytokine-driven hepcidin up-regulation leads to iron sequestration, resulting in further erythrocyte loss [13, 14]. Hence, anaemia due this inflammation, like iron deficiency anaemia, is among the most prevalent forms of anaemia worldwide [15].

To date, studies of anaemia in rural India have focused primarily on individual determinants of anaemia in isolation rather than looking at them jointly [16–25]. A significant gap remains in our knowledge of the relative contribution of various factors to the prevalence of anaemia among pregnant women in rural India using a conceptual framework [26, 27]. Evidence is also lacking specific to anaemia among pregnant women in rural Tamil Nadu state. Therefore, the objective of our study was to assess the prevalence of anaemia and to identify any coexisting determinants among exclusive biomass-using pregnant women in two rural sites in Tamil Nadu state.

## Methods

We conducted a cross-sectional analysis using baseline data from 799 pregnant women enrolled in the Indian sites of the Household Air Pollution Intervention Network (HAPIN) trial, a multi-centre randomised controlled trial implemented in four countries (Guatemala, India, Peru and Rwanda) to assess the health effects of a liquefied petroleum gas stove intervention. Details of the trial protocol [28] and rationale for the selection of study sites in India [29] are provided elsewhere. For the Indian site, study participants were identified from lists of pregnant women registered for routine antenatal care at health facilities in two districts in Tamil Nadu: Kallakurichi (formerly Villupuram) and Nagapattinam. A total of 799 pregnant women were enrolled between April 2018 and November 2019. Eligibility criteria included: aged 18–35 years, ultrasound-confirmed gestational age between 9 and 20 weeks and lived in a household that was using biomass as their primary cooking fuel.

Sociodemographic information was self-reported through surveys and included mother’s education, occupation, father’s education, household assets, access to a sanitation facility, access to safe drinking water, self-reported treatment of drinking water, and women’s autonomy in decision making. Additionally, enumerators asked participants to show them their hand washing facility and documented whether an observable designated hand-wash area was present. A household wealth index was calculated based on household asset ownership and classified into five categories [30]. Parity, birth spacing and details of consumption of micronutrient supplements were captured from medical records.

Haemoglobin (Hb) was measured from capillary blood using point-of-care testing (HemoCue^®^ 201) using standard procedure [31]. Field staff were trained and ensured that they followed standardized protocols for all capillary blood collection using standardized finger-prick procedures and device operation across the study sites.

WHO criteria for anaemia in pregnancy were used to categorize participants as normal (Hb ≥ 11 g/dL), mild (Hb 10.0–10.9 g/dL), moderate (Hb 7.0–9.9 g/dL), and severe anaemia (Hb < 7.0 g/dL) [30]. Anaemia was treated as a categorical variable in all analyses [32].

Height (Seca 213 stadiometer) and weight (Seca 876 digital scale) were measured by trained enumerators following standardised procedures in both study sites. Two readings of height were recorded to 0.1 cm and a third measurement was taken if the difference between the first two readings exceeded 1 cm. Similarly, two weight measurements were taken and recorded to 0.1 kg. If the difference between two measurements exceeded 0.5 kg, a third measurement was performed. Averages of the height and weight measurements were used to calculate body mass index (BMI) and categorised as normal (18.5 to 24.9 kg/m^2^), underweight (< 18.5 kg/m^2^), overweight (25 to 29.9 kg/m^2^), and obese (≥ 30 kg/m^2^) as per WHO classification [33].

Household food insecurity and dietary diversity were measured using the food insecurity experience scale (FIES) and minimum dietary diversity score for women (MDD-W), respectively [34]. FIES consists of eight questions on access to adequate food in the participant’s household in the past 30 days. Scores for the eight questions were summed and households were classified as being food secure (0), moderately food insecure (1 to 3), or severely food insecure (> 3). Data collection for the MDD-W involves an open recall of foods consumed in the previous day or night. For HAPIN, the MDD-W questionnaire was adapted to cover the previous 30 days, to align with the FIES. In MDD-W, ten food groups are included in the score: grains, white roots and tubers, and plantains; pulses (beans, peas, and lentils); nuts and seeds; dairy; meat, poultry, and fish; eggs; dark green leafy vegetables; other vitamin A-rich fruits and vegetables; other vegetables; and other fruits. A score of “1” was entered for those who reported a daily intake of each food group and others were given with the scores of “0”. The total is the sum of the scores for the above ten food groups, which could range from 0 to 10. Pregnant women with total scores ≥ 5 were categorised as achieving minimum dietary diversity and those with scores of < 5 were categorised as not achieving minimum dietary diversity. Physical activity of pregnant women was recorded using International Physical Activity Questionnaire–short form. The total metabolic equivalent of task (METs) per week was calculated. Other risk factors such as alcohol and tobacco use were self-reported.

### Statistical Analysis

The sample size was predetermined by the parent trial. All analyses were conducted using R software (R Foundation for Statistical Computing, Vienna, Austria). A descriptive analysis was conducted for both independent and dependent variables. Bivariate analysis was performed to identify independent variables significant at the *p* < 0.2 level, as shown in supplementary files which were subsequently included in the multivariable analysis. Binary logistic regression was used to identify variables associated with anaemia in general. Additionally, multinomial logistic regressions were employed to examine associations between the identified independent variables and two grades of anaemia: mild, and a combined group of moderate and severe anaemia. For the purpose of statistical analysis, moderate and severe anaemia were combined into a single category owing to the small number of pregnant women in the severe anaemia group (*n* = 9). There were only a few missing values in the covariates in the data set. Consequently, five records were excluded from the final analysis. Adjusted odds ratios (OR) are reported with 95% confidence intervals (CI).

## Results

Sociodemographic and obstetric details of pregnant women are shown in Table 1. The mean and standard deviation of participants age was 23.9 ± 3.8 years. Overall, 72% of study participants were either in the first or second lowest categories of the wealth index. Half of the pregnant women in Villupuram had neither any formal education nor completed primary school, whereas over 45% of the participants from Nagapattinam had completed secondary school and higher. Similar differences were also observed in father’s education. Agriculture (87%) was reported to be the major occupation of participants in Villupuram whereas almost all (97%) were homemakers in Nagapattinam. The mean gestational weeks at the time of assessment was 16 weeks. Around 50% were primi gravidae; 40% of the non-primi had children less than 3 years old.

**Table 1:** Socio Demographic and obstetric details of the pregnant women living in rural areas of two districts in Tamil Nadu, India

Description	Villupuram	Nagapattinam	Total	*p*-value
	*N* = 400	*N* = 399	*N* = 799	
Age (Years) [Mean ± SD]	23.0 ± 3.4	24.8 ± 4	23.9 ± 3.8	< 0.001
Education - self [n (%)]				
No formal education/Primary school incomplete	207(51.7)	78(19.5)	285(35.7)	0.002
High school incomplete	100(25.0)	127(31.8)	227(28.4)	
High school complete and other higher education	93(23.3)	194(48.6)	287(35.9)	
Education - spouse [n (%)]				
No formal education or Primary school incomplete	183(45.8)	121(30.3)	304(38)	< 0.001
Primary school complete	66(16.5)	56(14)	122(15.3)	
Secondary school complete or Vocational	49(12.3)	77(19.3)	126(15.8)	
Secondary school incomplete	52(13.0)	83(20.8)	135(16.9)	
Some college or university	47(11.8)	61(15.3)	108(13.5)	
Occupation-self [n%]				
Agriculture	337(84.3)	1(0.2)	338(42.3)	< 0.001
Home maker	45(11.3)	387(97)	432(54.1)	
Other	16(4.5)	11(2.8)	29(3.6)	
Wealth index [n (%)]				
Lowest	93(23.3)	86(21.6)	179(22.4)	< 0.001
Second Lowest	232(58)	169(42.4)	401(50.2)	
Middle	71(17.8)	105(26.3)	176(22.0)	
Second highest	4(1.0)	39(9.8)	43(5.4)	
Gestational Age at baseline (Weeks) [Mean ± SD]	16.6 ± 3.0	15.5 ± 3	16.1 ± 3	< 0.001
On vitamin supplements [n (%)]	390(97.5)	394(98.7)	784(98.1)	0.15
Primi gravidae [n (%)]	175(43.8)	217(54.4)	392(49.1)	0.03
Among multi gravidae [n (%)]	*N* = 225	*N* = 182	*N* = 407	P-value
Has children (< 3-year-old)	124(55.1)	70(38.5)	194(47.7)	0.01
Had C-Section	24(10.7)	71(39)	95(23.4)	< 0.001
Had stillbirth [n (%)]	7(3.1)	8(4.4)	15(3.8)	0.41
Had pre term birth [n (%)]	8(3.6)	4(2.2)	12(2.9)	0.39
Had abortion [n (%)]	32(14.2)	52(28.6)	84(20.6)	0.02
Chi-square or t-test were used appropriately as test of significance				

**Fig. 1:**
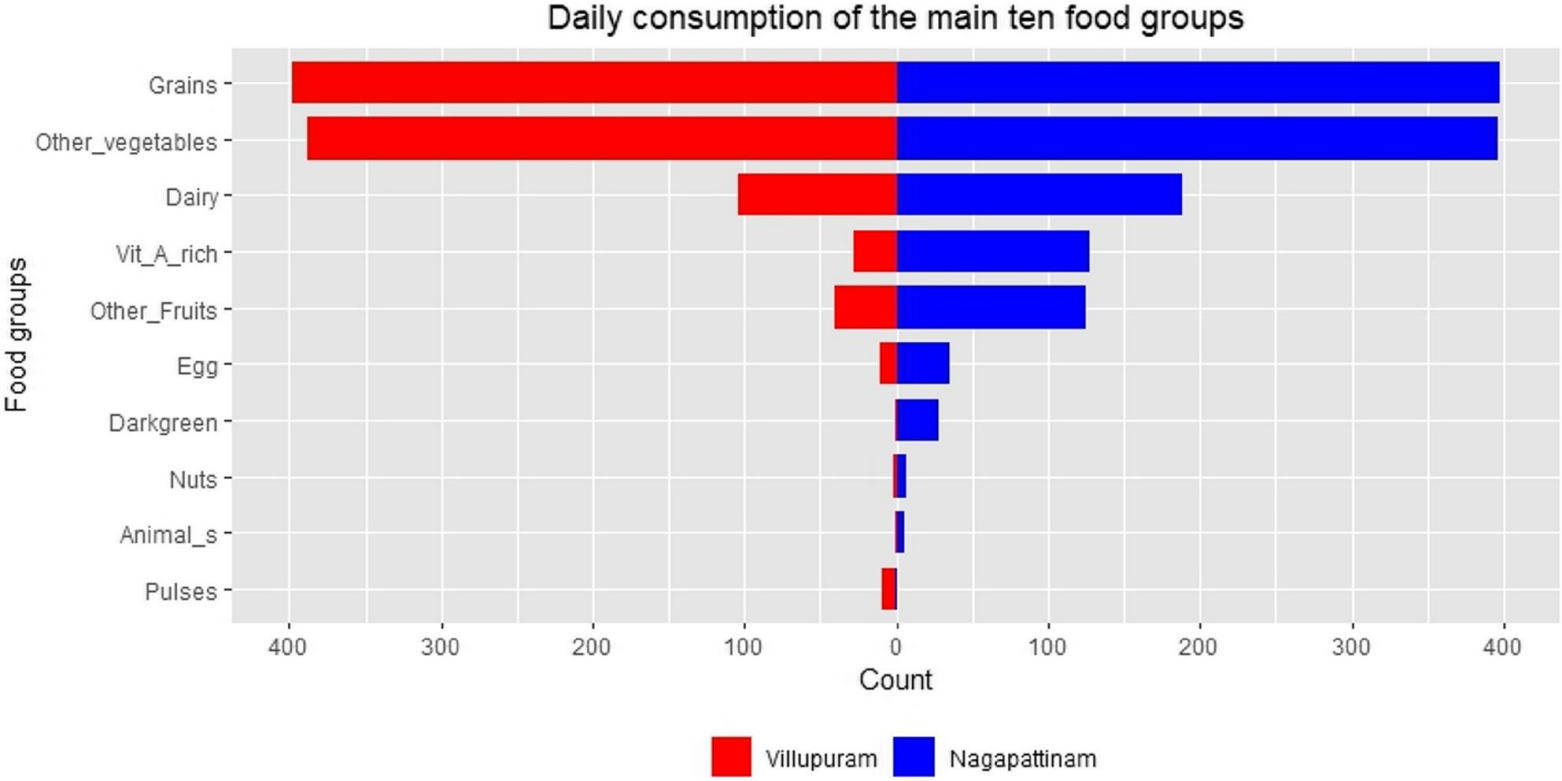
Comparison of daily intake of essential food groups between study sites

Table 2 shows anthropometry, diet and physical activity details of the study participants. The mean and standard deviation of BMI was 19.7 ± 3.2 kg/m^2^. Half of participants were in underweight category in Villupuram. Overall, 80% (Villupuram 72%; Nagapattinam 90%) reported to have no food insecurity. The majority of the participants met the pregnancy period physical activity recommendation of > 600METs/week. None of them reported any tobacco smoking or alcohol consumption. Overall, 89% of participants (Villupuram 99%, Nagapattinam 79%) did not achieve minimum dietary diversity, as shown in Fig. 1.

**Table 2:** Anthropometry, Diet and Physical activity details of the pregnant women living in rural areas of two districts in Tamil Nadu, India

	Villupuram	Nagapattinam	Total	*p*-value
Diet Diversity				
Not achieving minimum dietary diversity (< 5)	395(98.8)	317(79.1)	712(89.1)	<0.001
Achieved minimum dietary diversity (≥ 5)	5(1.3)	82(20.6)	87(10.9)	
Household food insecurity scale assessment				
No food insecurity (0)	286(71.5)	359(90)	645(80.7)	<0.001
Mild food insecurity present (1–3)	89(22.3)	25(6.3)	114(14.3)	
Moderate/Severe food insecurity present (> 3)	25(6.3)	11(2.8)	36(4.5)	
Physical activity				
Total METs per week Mean (SD)	1386(4851)	5832 (1680)	5040(5334)	< 0.001
Meeting PA recommendation in pregnancy (> 600METs per week)	351(87.8)	396(99.2)	747(93.5)	< 0.001
Anthropometry				
BMI (kg/m^2)^	18.8 ± 2.3	20.7 ± 3.6	19.7 ± 3.2	< 0.001
BMI categories				<0.001
Underweight (< 18.5 kg/m^2^)	199(49.8)	114(28.6)	313(39.2)	
Normal (18.5 to 24.9 kg/m^2^)	194(48.5)	235(58.9)	429(53.7)	
Overweight (25 to 29.9 kg/m^2^)	6(1.5)	44(11)	50(6.3)	
Obese (> 30 kg/m^2^)	1(0.3)	6(1.5)	7(0.9)	

Chi-square or t-test were used appropriately as test of significance

*BMI* Body Mass Index

Around 75% had access to safe drinking water and 98% reported following some method to treat their drinking water in Villupuram. On the other hand, 98% had access to safe drinking water and only 13% reported treating their drinking water in Nagapattinam. Open defaecation was common (Villupuram 93%, Nagapattinam 53%). Designated hand wash facility was observed in most of the houses in Villupuram but in only 18% of the houses in Nagapattinam, as shown in Table 3.

**Table 3:** Details on water, sanitation and hand hygiene practices among the pregnant women living in rural areas of two districts in Tamil Nadu, India

Characteristics	Villupuram	Nagapattinam	Total	*p*-value
Access to safe drinking water	303(75.8)	390(97.7)	693(86.7)	< 0.001
Water treatment method used	393(98.3)	51(12.8)	444(55.6)	< 0.001
No observable designated hand-wash area	2(0.8)	287(71.9)	289(36.2)	< 0.001
Toilet - No facility (Bush/Fields)	372(93)	211(52.9)	583(73)	< 0.001
Involved in decision making in				
Major Purchase	191(47.8)	181(45.3)	372(46.6)	0.52
Daily Purchase	194(48.6)	181(45.3)	375(46.9)	0.41
Visiting the relative’s and other places	180(45)	172(43.1)	352(44.1)	0.62

Chi-square or t-test were used appropriately as test of significance

**Fig. 2:**
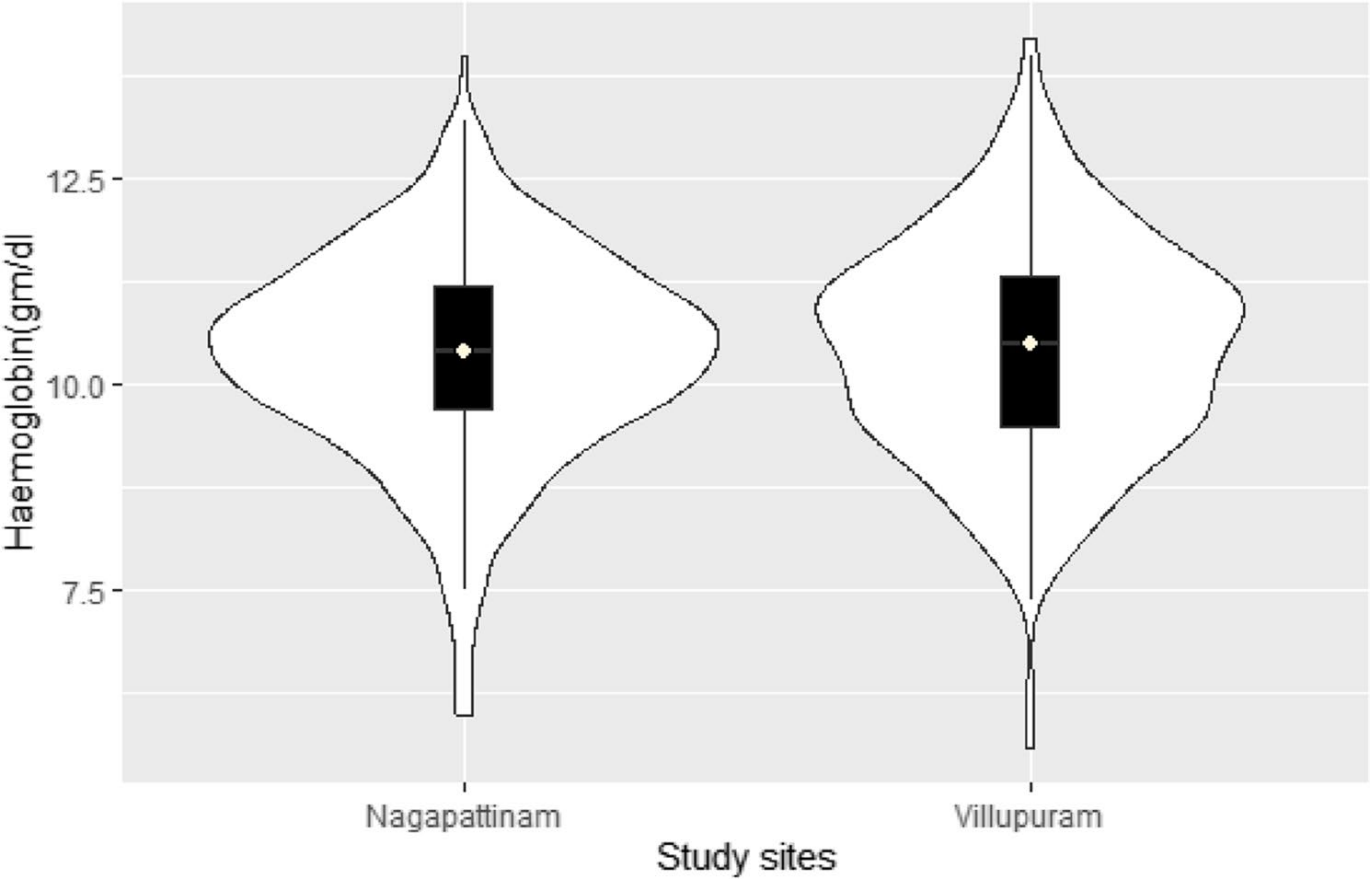
Distribution of Haemoglobin level in pregnant women by study site

**Fig. 3:**
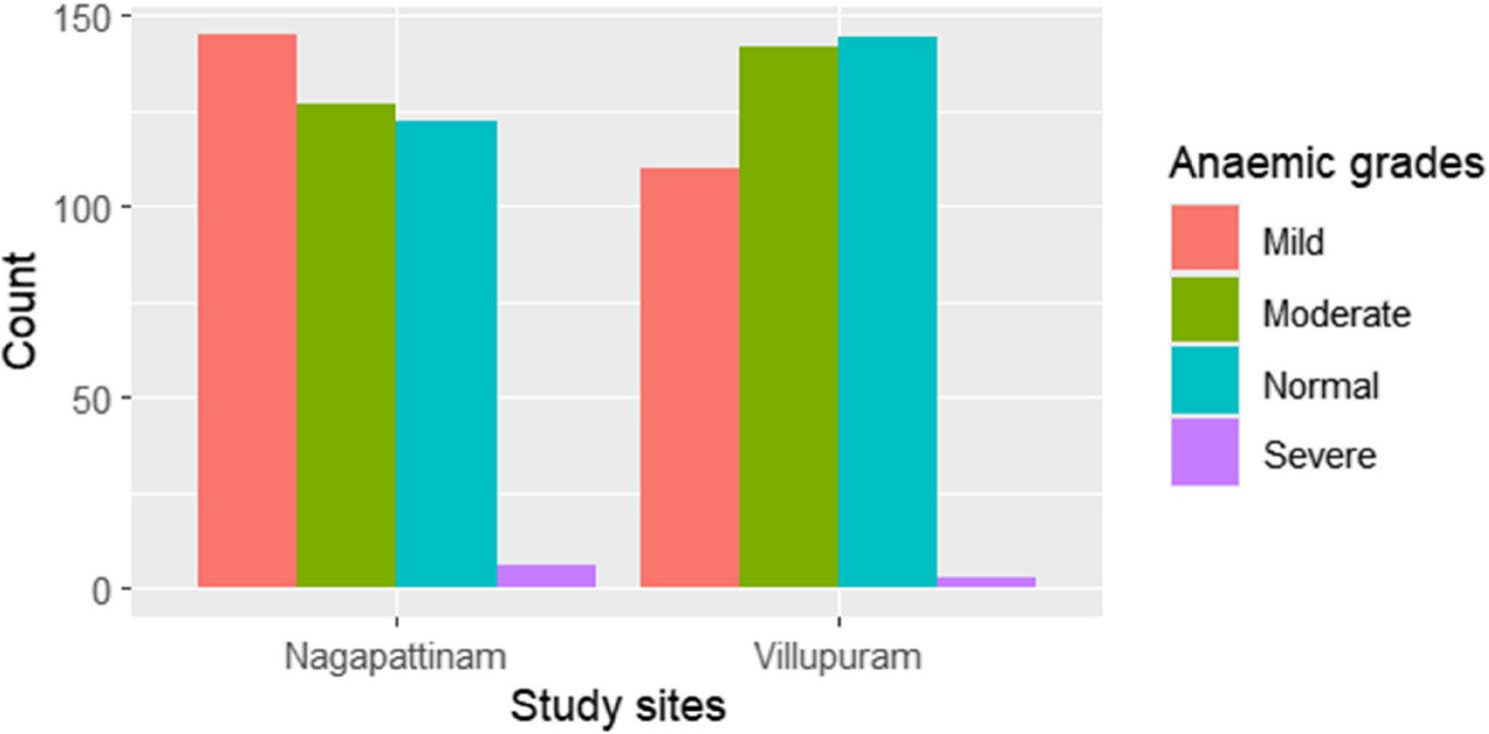
Prevalence of different grades of anaemia in pregnant women across the study sites

Figure 2 shows that the mean Hb levels (10.4 ± 1.3gm/dl; p-value = 0.06) were similar between the study sites. The overall prevalence of anaemia was 66.7% (95% CI: 63.3–70.0%), and the comparisons by site and severity are shown in Fig. 3. In Villupuram, 27.5% of participants were mildly anaemic, 35.8% moderately anaemic and 0.8% severely anaemic. Similarly, from Nagapattinam, 36.3% had mild, 31.3% had moderate and 1.8% had severe anaemia (*p* = 0.03).

Results of uni-variable associations with any form of anaemia and that across two grades (i.e., mild and a combined group of moderate and severe anaemia) is illustrated in supplementary tables ST1 to ST4.

Results from binary logistic regression for total anaemia, unadjusted (model-1) and adjusted for study sites (model-2), are shown in Table 4. Multi-gravidae [OR (CI; p-value)], [1.6 (1.1 to 2.28; *p* = 0.03)] and underweight [1.5 (1.1 to 2.03); *p* = 0.04] had higher odds of anaemia. Pregnant women in overweight or obese was associated with lower odds of anaemia [0.5 (0.3 to 1.0); *p* = 0.02]. After adjusting for study sites, two additional variables, no observable designated hand wash facilities in their dwelling [1.8 (1.1 to 2.9); *p* = 0.02] and having children > 3 years old [1.6 (1.0 to 2.4); *p* = 0.04] were significantly associated with anaemia. The wealth index, gestational age, self-reported treatment of drinking water, minimum dietary diversity score, food insecurity, and occupation were not associated with total anaemia in either model.

**Table 4:** Results of Binary Logistic Regression analysis-normal versus any form of anaemia as dependent variable

Variables	Odds ratio with (95% CI)	
	Model-1	Model-2
GA (weeks) at baseline	1.1(1.0 to 1.1)	1.1(1.0 to 1.1)
An observable designated hand-washing area	Ref	Ref
No	1.5 (0.9 to 2.4)	1.8 (1.1 to 3)^
Water treatment method used-Yes	Ref	Ref
No	1.2 (0.7 to 2)	1.8 (1 to 3.3)
Household food insecurity scores	1 (0.9 to 1.2)	1.1 (0.9 to 1.1)
Wealth indices- Lowest	Ref	Ref
Second Lowest	1.1 (0.7 to 1.6)	1.1 (0.7 to 1.5)
Middle	0.8 (0.5 to 1.3)	0.8 (0.5 to 1.2)
Second highest	2.0 (0.9 to 4.7)	2.2 (0.9 to 5.1)
Minimum diet diversity score	1 (0.9 to 1.2)	1.1 (0.9 to 1.2)
BMI –Normal	Ref	Ref
Underweight	1.5 (1.1 to 2)*	1.4 (1 to 2)^
Overweight & obesity	0.5 (0.3 to 0.9)*	0.5 (0.3 to 1)^
Primi gravida -Yes	Ref	Ref
No	1.5 (1.1to 2.3)*	1.6 (1.1 to 2.4)^
Occupation-Agriculture	Ref	Ref
Home maker	1.04 (0.6 to 1.7)	1.6 (0.9 to 3.1)
Others	1.3 (0.6 to 3.1)	1.5 (0.6 to 3.6)
Having child < 3 years -Yes	Ref	Ref
No	1.5(1.0 to 2.3)	1.6 (1.0 to 2.4)^

Occupation -Other (include those working in industries, health sector, self employed and unemployed)

Model-1 is unadjusted for study sites

Model-2 is adjusted for study sites

Having child < 3years old is reverse coded and considered as reference group

*GA* Gestational age, *BMI* Body Mass Index

* Represents the variables with statistical significance at 0.05 level for model-1

^ Represents the variables with statistical significance at 0.05 level for model-2

Factors responsible for mild, moderate and severe (combined together) anaemia are shown in Table 5. Being in the second highest category of the wealth index was associated with mild anaemia [2.9 (1.2 to 7.2); *p* = 0.03)], but not with moderate and severe anaemia.

The pregnant women who were classified as being underweight had [1.6 (1.1 to 2.4); *p* = 0.08)] higher odds of developing moderate and severe anaemia compared to those who had normal BMI. No observable designated hand-wash area [2.0 (1.1 to 3.6); *p* = 0.02] was significantly associated with moderate and severe anaemia after adjusting for study sites.

**Table 5:** Multinomial regression analysis- normal vs. mild, moderate and severe grades of anaemia

Variables	Odds ratio (95% confidence interval)			
	Mild Anaemia		Moderate & Severe Anaemia	
	Model − 1	Model-2	Model − 1	Model-2
Age (years)	1.0 (1 to 1.1)	1.0 (1 to 1.1)	1.0 (0.9 to 1)	1.0 (0.95 to 1.1)
Minimum dietary diversity	1.1 (1 to 1.3)	1.1(0.9 to 1.3)	1.0 (0.8 to 1.2)	1 (0.9 to 1.2)
Education = Higher education (ref) No formal education/Primary school incomplete	1(0.6 to 1.6)	1(0.6 to 1.5)	1.2 (0.8 to 1.9)	1.2 (0.7 to 1.8)
Primary school complete or Secondary school incomplete	0.8 (0.5 to 1.3	0.8 (0.5 to 1.3)	1.6 (1 to 2.5)	1.5 (1 to 2.4)
Occupation – Agriculture (ref) Home maker	1.4 (0.5 to 4.4)	1.4 (0.4 to 4.3)	0.5 (0.2 to 1.2)	0.4 (0.2 to 1.1)
Other	1.3 (0.5 to 3.9)	1.5 (0.5 to 4.6)	0.4 (0.2 to 1.4)	0.8 (0.3 to 2.1)
BMI- Normal	Ref	Ref	Ref	Ref
Underweight	1.0 (0.7 to 1.5)	1.0 (0.7 to 1.5)	1.6 (1.1 to 2.4)*	1.6 (1.1 to 2.3)^
Overweight & obese	0.7 (0.4 to 1.4)	0.7 (0.4 to 1.4)	0.3 (0.2 to 0.8)*	0.3 (0.2 to 0.8)^
Water treatment method used -Yes	Ref	Ref	Ref	Ref
No	1.4 (0.8 to 2.5)	1.6 (0.82 to 3.2)	1 (0.6 to 1.8)	1.6 (0.8 to 3.4)
Having child < 3years old -Yes	Ref	Ref	Ref	Ref
No	1.1 (0.7 to 1.6)	1.1 (0.7 to 1.7)	1.2 (0.8 to 1.8)	1.2 (0.8 to 1.8)
An observable hand-wash facility- Yes	Ref	Ref	Ref	Ref
No	1.5 (0.9 to 2.5)	1.6(0.9 to 2.8)	1.6 (0.9 to 2.7)	2.0(1.1 to 3.6)^
Open defaecation -Yes	Ref	Ref	Ref	Ref
No	0.21(0.02 to2.3)	0.23(0.02 to 2.5)	0.27(0.02 to2.9)	0.28(0.03 to 3.1)
Wealth indices – Lowest	Ref	Ref	Ref	Ref
2nd higher	2.9 (1.2 to 7.2)*	2.9 (1.2 to 7.4)^	1.3(0.5 to 3.6)	1.4(0.5 to 3.9)
Middle	0.8 (0.5 to 1.4)	0.8 (0.5 to 1.4)	0.8 (0.5 to 1.3)	0.8(0.5 to 1.3)
2nd lower	1.1 (0.7 to 1.7)	1.1 (0.7 to 1.7)	1 (0.7 to 1.7)	1(0.7 to 1.6)

Model-1 is unadjusted for study sites

Model-2 is adjusted for study sites

Occupation -Other (includes those working in industries, health sector, self-employed and unemployed)

Having children < 3years old is reverse-coded and considered as reference group

*BMI* Body Mass Index

* Represents the variables with statistical significance at 0.05 level for model-1

^ Represents the variables with statistical significance at 0.05 level for model-2

## Discussion

In this cross-sectional study of 799 rural pregnant women (9 to 20 weeks gestation) exclusively using biomass fuel for cooking in two districts of Tamil Nadu, India, we found that 66.7% were anaemic, a prevalence that is substantially higher than the NFHS-5 state average for Tamil Nadu. Our results indicate that low BMI, no observable hand washing facility, being multigravida, and, among the multi paras, having children more than 3 years old were all significantly associated with anaemia in this population. Inadequate dietary diversity, limited access to safe drinking water and unimproved sanitation are highly prevalent in these communities but were not found to be associated with anaemia in our models. These null results may reflect a lack of heterogeneity, as nearly all participants had limited access to diverse diets and improved sanitation. Alternatively, the results may be attributable to the dominant influence of biomass-related inflammation, which could be overwhelming the other factors.

We observed no significant association between wealth and anaemia, except for among participants in the second highest category (disproportionately lower in distribution of the study participants), suggesting that anaemia prevalence remains broadly similar across all wealth categories among our study population. This can be attributed to the lack of heterogeneity in the economic stream of the study participants and also to the possibility of sharing a common aetiology of inflammation-induced anaemia due to cooking with biomass. Being in the overweight and obese categories based on BMI appeared to have a protective effect against anaemia, which may be attributable to the disproportionately smaller numbers within these BMI groups and, potentially, to the role of improved nutrient intake in mitigating inflammation.

Similar studies conducted among pregnant women in rural settings of Tamil Nadu have found varying prevalences of anaemia. A study conducted by Abiselvi et al. among 270 pregnant women in Tamil Nadu in 2015 observed an anaemia prevalence of 41.5% [16]. A different study conducted among 1290 pregnant women in a similar rural setting in 2018 showed a prevalence of 62% [24]. The differences could be attributed to variability in sampling frame, the pregnancy period assessment, haemoglobin estimation methods used, and the mixed socioeconomic status in the study settings. The prevalence of anaemia of the current study finding is more comparable with that of a study of 21,473 pregnant women in 2019–2020 that showed a prevalence of 70% [35]. In that study, the study population was women attending antenatal care at a government hospital whose catchment area included Villupuram district, potentially overlapping with our study population.

The current study observed the classical risk factors of anaemia such as lack of education, limited access to safe drinking water, and poor sanitation/hygiene practice are highly prevalent in this cohort of pregnant women who use biomass for cooking. These are consistent with the findings of Sunuwar et al., who examined factors associated with anaemia across seven South and Southeast Asian countries [36]. While the Sunuwar study examined a larger group of women of reproductive age in high-burden Asian countries, the common predictors remain clear.

Our results can also be compared to our previous research among a rural-urban cohort of women in Tamil Nadu. The rural cohorts who were part of that (2010–2012) study had a much higher prevalence of mild (65% versus 31.9% in our study) and slightly lower moderate (30% versus 33.9% in our study) anaemia [37]. A modest decline in the anaemia prevalence in the state was observed till 2016 that could be attributed to the outreach and increased utilisation of national programme [38].

A comparative assessment between biomass stove users with that of clean fuel users in rural pregnant women in India showed a higher relative risk for mild (1.4) and moderate to severe (1.8) anaemia [11] among the biomass users, indicating the possible role of inflammation in the aetiology of anaemia. Hence, the higher prevalence observed in the current study may be attributed to the additive effect of low socioeconomic conditions and use of unclean fuel. Similar findings were reported in a recent study conducted in China [39].

Within the current study, regarding the study enrollment process and the high target rate for pregnant women to be enrolled, the time gap between antenatal registration and haemoglobin assessment rarely exceeded one month. This indicates that it is highly unlikely that the iron and folic acid supplementation received during that period influenced the estimated prevalence of anaemia. Based on this finding, prioritising targeting adolescent girls and young women must be a top priority moving forward.

Underweight pregnant women with high rates of moderate anaemia and low dietary diversity dominated our study population. Inadequate dietary intake was likely compounded by exposure to household air pollution from unclean biomass fuels, which further disrupts iron metabolism and red cell health. Addressing this dual burden requires coordinated efforts to expand clean fuel access alongside strengthening local agriculture and public distribution systems to increase diet diversity. A combined focus on nutrition and clean energy provision is essential to improve maternal nutritional status and to reduce anaemia in such rural communities [40].

Across our two study sites, participants in Nagapattinam had a relatively better economic condition, higher educational attainment, higher dietary diversity, and lesser prevalence of underweight, yet they still had a high prevalence of anaemia. Despite their better economic conditions, a lack of treatment of drinking water and lack of hand washing facilities were observed in this site. Our results highlight a need for identification of such poor performing communities, looking beyond socioeconomic status, with respect to maternal and child malnutrition and its associated determinants.

An important strength of our study is the contribution of data on anaemia prevalence among pregnant women in two rural districts of Tamil Nadu. We utilized data from HAPIN trial that followed standard protocols with an adequate sample size while considering many factors known to contribute to anaemia. At the same time, our study also has some limitations. The cross-sectional design limits causal inference. As the primary study required all participants to use biomass as their primary cooking fuel, we were unable to quantify associations between fuel type and anemia within a counterfactual framework using the baseline data. HemoCue devices are widely used for community studies due to their portability and immediate results in resource-limited areas, where venous sampling is often impractical. However, the findings require careful interpretation, as measurements can be subject to random error. In addition, capillary haemoglobin concentrations may be marginally lower than venous measurements, potentially resulting in an overestimation of anaemia prevalence.

While our study population is richly characterized, some data limitations exist. For example, the use of a monthly rather than a 24-hour recall for MDD-W may have contributed to an apparent overestimation of diet diversity score. However, it should be noted that the overall proportion of pregnant women in this study population achieving minimum dietary diversity was minimal, with only 10.9%. We did not collect data on the duration of consumption of iron supplementation, biomarkers to understand the other aetiologies of anaemia (e.g., micronutrient deficiencies, infection, inflammatory markers and genetic disorders), or on intake of enhancers and inhibitors of iron absorption. In addition, inadequate access to health services is considered to be an underlying cause of anaemia, but given that our study population was drawn from pregnant women who were registered for routine antenatal care at health facilities, we were not able to include this factor in our models. Finally, while a notable strength of our study is its focus on a high-risk group of biomass users, this simultaneously limits the generalizability of our results.

Further research targeting reproductive-age women, with cleaner fuel interventions, is necessary to estimate personal exposure to PM_2.5_ and carbon monoxide, and to establish the causal impact of household air pollution on anaemia. The current study findings reveal that the existing burden of pregnancy anaemia in a subset of populations using biomass stoves are higher than that of the NFHS estimates for Tamil Nadu. Furthermore, the identified determinants of anaemia are modifiable and offer an opportunity for improvement with enhancement of socioeconomic components, with sustained investment in clean fuel access and health interventions.

## Supplementary Information

Supplementary Material 1

## Data Availability

The data that support the findings of this study are available upon request to the corresponding author.
