# Supplementary material for: Prevalence and determinants of anaemia among pregnant women using biomass fuel in rural Tamil Nadu: a cross-sectional study
Source: BMC Pregnancy Childbirth. 2026 Mar 27;26:555. doi: 10.1186/s12884-026-08999-1 (PMC13188275; doi:10.1186/s12884-026-08999-1)
Supplement: Supplementary file 1 — Supplementary Material 1 [file 12884_2026_8999_MOESM1_ESM.docx]

**Supplementary tables**

ST-1 Socio-demographic details of rural pregnant women from two districts from Tamil Nadu state across different anaemic groups

| Description | Normal  N=266 | Mild form  N=255 | Moderate & Severe form  N=278 | Normal vs  Mild  vs Moderate  p-value | Any form of anaemia  N=533 | Normal vs any form of anaemia  p-value |
| --- | --- | --- | --- | --- | --- | --- |
| Age (Years) [Mean ± SD] | 24±3.8 | 24.3±3.8 | 23.7±3.8 | 0.19 | 23.9±3.8 | 0.88 |
| Education - self [n %]  No formal education/ Primary school Incomplete | 101(38) | 89(34.9) | 95(34.2) | 0.03 | 184(34.5) | 0.45 |
| High school incomplete | 69(25.9) | 61(23.9) | 97(34.9) |  | 158(29.6) |  |
| High school complete and other higher education | 96(36.1) | 105(41.2) | 86(30.9) |  | 191(35.8) |  |
| Education - spouse [n%]  No formal education or Primary school incomplete | 102(38.3) | 93(36.5) | 109(39.2) | 0.8 | 202(37.9) | 0.84 |
| Primary school complete | 44(16.5) | 38(14.9) | 40(14.4) |  | 78(14.6) |  |
| Secondary school complete or Vocational | 37(13.9) | 41(16.1) | 48(17.3) |  | 89(16.7) |  |
| Secondary school incomplete | 43(16.2) | 41(16.1) | 51(18.3) |  | 92(17.3) |  |
| Some college or university | 38(14.3) | 41(16.1) | 29(10.4) |  | 70(13.1) |  |
| Occupation[n%]  Agriculture | 126(47.4) | 96(37.6) | 116(41.7) | 0.06 | 212(39.8) | 0.12 |
| Household work | 131(49.2) | 153(60) | 148(53.2) |  | 301(56.5) |  |
| Other | 9(3.4) | 6(2.4) | 14(5.1) |  | 20(3.8) |  |
| National Wealth indices [n %]  Lowest | 58(21.8) | 53(20.8) | 68(24.5) | 0.02 | 121(22.7) | 0.15 |
| Second Lowest | 131(49.2) | 125(49) | 145(52.2) |  | 270(50.7) |  |
| Middle | 68(25.6) | 53(20.8) | 55(19.8) |  | 108(20.3) |  |
| Second Highest | 9(3.4) | 24(9.4) | 10(3.6) |  | 34(6.4) |  |

ST-2 Gestational details of the rural pregnant women from two districts of Tamil Nadu state based on their anaemic status

| Description | Normal N=266 | Mild form  N=255 | Moderate/Severe form  N=278 | Normal vs  Mild  vs Moderate  p-value | Any form of anaemia  N=533 | Normal vs.any form of anaemia  p-value |
| --- | --- | --- | --- | --- | --- | --- |
| Gestational Age at baseline (Weeks) [Mean ± SD] | 15.7±3.1 | 16±3 | 16.4±3 | 0.38 | 16.2±3 | 0.04 |
| Primi gravidae [n %]  Multi gravidae [n %] | 137(51.5)  129(48.5) | 122(47.8)  133(52.2) | 133(47.8)  145(52.2) | 0.62 | 255(47.8)  278(52.2) | 0.37 |
| Birth Spacing [n %]  Has children (<3-year-old) | 70(54.3) | 61(45.9) | 63(43.4) | 0.18 | 124(44.6) | 0.05 |

ST-3 Anthropometry, diet and physical activity details of rural pregnant women from two districts of Tamil Nadu state across different anaemic groups

|  | Normal  N=266 | Mild form N=255 | Moderate/Severe form  N=278 | Normal vs  Mild  vs Moderate  p-value | Any form of anaemia  N=533 | Normal vs any form of anaemia  p-value |
| --- | --- | --- | --- | --- | --- | --- |
| Diet Diversity categories  Not achieving minimum dietary diversity (<5) | 241(90.6) | 219(85.9) | 252(90.6) | 0.13 | 471(88.4) | 0.2 |
| Achieved minimum dietary diversity (≥5) | 25(9.4) | 36(14.1) | 26(9.4) |  | 62(11.6) |  |
| Household food insecurity scale assessment  No food insecurity (0) | 217(81.6) | 214(83.9 | 214(77) | 0.89 | 428(80.3) | 0.23 |
| Mild food insecurity present (1-3) | 35(13.2) | 27(10.6) | 52(18.7) |  | 79(14.8) |  |
| Moderate/Severe food insecurity present (>3) | 13(4.9) | 12(4.7) | 11(4.0) |  | 23(4.3) |  |
|  |  |  |  |  |  |  |
| Meeting PA recommendation in pregnancy (>600METs per week) | 247(92.9) | 235(92.2) | 265(95.3) | 0.3 | 500(93.8) | 0.65 |
| Anthropometry  BMI (kg/m^2^) | 20±3.3 | 20.1±3.4 | 19.2±2.8 | 0.001 | 19.6±3.1 | 0.12 |
| BMI categories  Underweight ((<18.5) | 94(35.3) | 86(33.7) | 133(47.8) | 0.002 | 219(41.1) | 0.22 |
| Normal ((18.5 to 24.9) | 147(55.3) | 146(57.3) | 136(48.9) |  | 282(52.9) |  |
| Overweight (25 to 29.9) | 22(8.3) | 20(7.8) | 8(2.9) |  | 28(5.3) |  |
| Obese (>30) | 3(1.1) | 3(1.2) | 1(0.4) |  | 4(0.8) |  |

PA- Physical Activity

BMI - Body Mass Index

ST-4 Details on water, sanitation and hand hygiene practices observed in rural pregnant women from two districts of Tamil Nadu state across different anaemic groups

|  | Normal  N=266 | Mild form  N=255 | Moderate/Severe form  N=278 | Normal vs  Mild  vs Moderate | Any form of anaemia  N=533 | Normal vs any form of anaemia p-value |
| --- | --- | --- | --- | --- | --- | --- |
| Access to safe drinking water sources | 232(87.2) | 225(88.2) | 236(84.9) | 0.5 | 461(86.5) | 0.83 |
| Water treatment method used | 103(38.7) | 130(51) | 122(43.9) | 0.12 | 252(47.3) | 0.04 |
| No observable hand wash facility | 78(29.3) | 105(41.2) | 106(38.1) | 0.02 | 211(39.6) | 0.02 |
| Open defaecation | 194(72.9) | 174(68.2) | 215(77.3) | 0.06 | 389(73) | 0.53 |
| Involved in decision making on |  |  |  |  |  |  |
| Major Purchase | 120(45.1) | 116(45.5) | 136(48.9) | 0.62 | 252(47.3) | 0.59 |
| Daily Purchase | 127(47.7) | 120(47.1) | 128(46) | 0.9 | 248(46.5) | 0.76 |
| Visiting-relatives &other places | 111(41.7) | 111(43.5) | 130(46.8) | 0.49 | 241(45.2) | 0.37 |
